# Histologic Reappraisal and Evaluation of MLH1 Protein Expression in Sessile Serrated Lesions of the Proximal Colon

**DOI:** 10.1155/grp/5970839

**Published:** 2025-12-19

**Authors:** Priscilla de Sene Portel Oliveira, Miriam Aparecida da Silva Trevisan, Rita Barbosa de Carvalho, Rita de Cássia Perina Martins, João José Fagundes, Claudio Saddy Rodrigues Coy

**Affiliations:** ^1^ Department of Surgery, School of Medical Sciences, Campinas State University, São Paulo, Brazil, unicamp.br; ^2^ Department of Pathological Anatomy, School of Medical Sciences, Campinas State University, São Paulo, Brazil, unicamp.br; ^3^ Independent Researcher, São Paulo, Brazil

## Abstract

**Introduction:**

Changes in nomenclature and in the criteria for histological diagnosis of the serrated lesions have occurred over the years. Some of these lesions, the sessile serrated lesions (SSLs), progress to adenocarcinoma via suppression of the *MLH1* gene, an important carcinogenic pathway.

**Objective:**

Evaluate the frequency of reclassification in histological diagnosis from hyperplastic polyps (HPs) to SSL after reappraisal using the 2019 World Health Organization (WHO) classification, to determine the occurrence of previously undiagnosed dysplasia and to study the expression of the *MLH1* protein in SSLs and SSLDs.

**Methodology:**

Lesions with histological diagnosis of HP, SSL, and SSLD resected by colonoscopies performed between 2005 and 2015 located in the proximal colon were studied. All HPs were submitted for histological review by two pathologists (Examiners 1 and 2), and a third experienced pathologist (Examiner 3) made the final decision when the other examiners did not agree. Interobserver agreement was analyzed. *MLH1* protein expression was assessed by immunohistochemistry in lesions diagnosed as SSL and SSLD before and after reappraisal. These lesions were reviewed again for missed dysplasia.

**Results:**

A total of 308 lesions were assessed being 287 with the initial diagnosis of HP and 21 SSL. Thirty‐eight (13.3%) lesions with an initial diagnosis of HP had their diagnosis reclassified to SSL. No dysplasia was found. There was a moderate agreement (Kappa 0.52) between Examiners 1 and 2 regarding the diagnosis of SSL. Between Examiners 1 and 3, there was no agreement (Kappa −0.19), and between Examiners 3 and 2, the agreement was poor (Kappa 0.13). All 38 lesions analyzed by immunohistochemistry had *MLH1* expression.

**Conclusion:**

Changes in diagnosis from HP to SSL occurred in 13.3%. No dysplasia or lack of MLH1 expression was observed.

## 1. Introduction

More than three decades after the publication of the study by Longacre and Fenoglio‐Preiser [1], serrated lesions remain difficult to diagnose. Although the pathway of carcinogenesis is well known, changes in nomenclature and histological criteria over the years have made it challenging to correctly identify these lesions [2–8].

The latest change occurred in 2019 by the World Health Organization (WHO) that changed the nomenclature of sessile serrated adenomas/polyps (SSAs/Ps) to sessile serrated lesions with and without dysplasia (SSLDs and SSLs, respectively) [8]. It also established that the occurrence of large unequivocal architectural distortion in a crypt is sufficient for the diagnosis, thus differentiating them from hyperplastic polyps (HPs).

The HPs located in the descending colon, sigmoid colon, and rectum are usually ≤ 5 mm and appear as discrete mucosal elevations when identified during colonoscopy. The HPs located in the remaining segments of the colon are similar to the SSLs in their macroscopic appearance: sessile to flat, pale, poorly defined and usually covered with a mucus cap. The diagnosis of these lesions is based exclusively on histological examination [8, 9].

The correct identification of SSLs has clinical importance since it is associated with carcinogenesis of the serrated pathway, which is considered to account for 15%–40% of sporadic adenocarcinomas [8–11]. This pathway involves the mutation of the *BRAF* gene, hypermethylation of DNA regions, and silencing of the *MLH1* gene, at which point rapid progression to adenocarcinoma occurs [9–13]. *MLH1* gene silencing occurs in all advanced lesions with dysplasia, known as SSLDs; therefore, analysis of the expression of the *MLH1* gene is proposed when there are doubts about the presence of dysplasia [14, 15].

It is necessary to better understand the evolution of SSLs to correctly assign a prognosis and thus define strategies for the management of patients with this type of lesion. The aims of this study were to evaluate the occurrence of changes in histological diagnosis from HP (before reappraisal) to SSL (after reappraisal) using the 2019 WHO classification and to determine the occurrence of previously undiagnosed dysplasia and expression of the MLH1 protein in SSLs and SSLDs.

## 2. Methods

### 2.1. Study Design

This was a retrospective study comprising the reappraisal of the histological and immunohistochemical reaction of lesions of the proximal colon (considered from the cecum to the splenic flexure) resected by colonoscopy that had been previously diagnosed as SSLs and HPs by histological examination. All tests were performed at the Gastrocentro, State University of Campinas, UNICAMP, São Paulo, Brazil, between January 1, 2005, and December 31, 2014.

Lesions of the proximal colon who received the histological diagnosis of HPs over the 10‐year study period were submitted for histological review. This review was performed by two pathologists with experience in lesions of the gastrointestinal tract (Examiners 1 and 2), and a third experienced pathologist (Examiner 3) analyzed lesions that received different diagnoses from the first two reviewers and made the final decision. The examiners were blinded to each other′s diagnoses, and the criteria used to diagnose SSLs and SSLDs in the histological review were those established by WHO in 2019 [8].

After review, the demographic and morphological data of all patients whose lesions′ diagnoses were changed from HP to SSL or SSLD, as well as those who received an initial diagnosis of SSL or SSLD in the first examination, were assessed (including age, sex, smoking status, history of colorectal cancer, size, localization and morphology of the SSLs and SSLDs, and presence of synchronous epithelial lesions). MLH1 protein expression was assessed by immunohistochemistry in lesions diagnosed as SSL or SSLD before or after reappraisal; these lesions were also reviewed by Examiner 3.

Lesions were divided into Group 1 (initial diagnosis of HP, SSL, or SSLD) and Group 2 (diagnosis of SSL or SSLD after review).

### 2.2. Participants

Patients with a lesion of the proximal colon (considered from the cecum to the splenic flexure) with a histological diagnosis of HP, SSL, or SSLD, which was completely resected by colonoscopy, were included in the study. All patients underwent colonoscopy at the Gastrocentro of UNICAMP, a public health unit specializing in gastrointestinal diseases. Patients with serrated polyposis syndrome, familial adenomatous polyposis, Lynch syndrome, whose colonoscopy was performed to resect a residual lesion, who had an inaccurate description of the lesion, whose colonoscopy and histology reports differed regarding the number of lesions, and whose examination did not reach the cecum or had an unsatisfactory bowel cleansing were excluded.

### 2.3. Variables

Age, sex, smoking status, history of colorectal cancer, and presence of synchronous lesions throughout the entire extent of the colon and rectum (adenomas, other serrated lesions, and adenocarcinomas) of patients with lesions diagnosed as SSLs or SSLDs were analyzed. Lesion size was defined as < 10 and ≥ 10 mm, and lesion morphology was described according to the Paris Classification [16]. Locations were considered by dividing the right colon into cecum and ascending and transverse colon.

The agreement between the examiners who conducted the histological reappraisal was analyzed (between Examiners 1 and 2, between Examiners 1 and 3, and between Examiners 2 and 3).

### 2.4. Immunohistochemistry

The expression of the MLH1 protein in lesions diagnosed as SSLs or SSLDs was evaluated by Examiner 3 by immunohistochemistry (Dako brand material, Batch 11100169, Clone ES05, dilution: pure). The paraffin block was separated and sectioned, and the specimens were fixed on slides with adhesive material. The next steps were antigen retrieval using the wet heat method, incubation with antibodies at 4°C for 12 h or at 37°C for 2 h, revelation using the peroxidase polymer‐conjugated secondary antibody system, staining with 3,3‐diaminobenzidine and counterstaining with hematoxylin, and validation of the reaction using external and internal controls.

The diagnosis of loss of protein expression was considered in cases wherein no staining was observed along the entire length of the lesion, that is, from the base to the apex of the crypt at least one region of the lamina.

### 2.5. Statistical Analysis

The Kappa method [17] was used to assess the agreement between the examiners. The chi‐squared test was used to analyze categorical variables, and the significance level was set at 5%. Statistical analysis was performed using Microsoft Office Excel software.

### 2.6. Ethics

The study was approved by the local Ethics Committee (CAAE 80599717.0.0000.5405/Opinion Number 2.459.313).

## 3. Results

### 3.1. General Population

A total of 251 patients were included in the study, with a mean age of 62 (20–93) years, being 142 (51.3%) males. Considering the diagnosed lesions as SSLs in the first histological analysis, concomitant lesions resected in the same colonoscopy, and patients who underwent more than one exam throughout the study period, a total of 308 lesions were assessed. In the first histological analysis, 21 and 287 lesions were diagnosed as SSLs and HPs, respectively.

### 3.2. Histological Reappraisal

**Figure 1:**
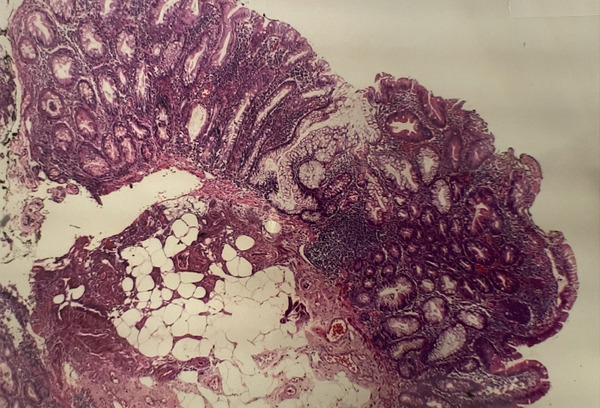
Sessile serrated lesion without dysplasia. The presence of a crypt with an enlarged, L‐shaped base and serration in other crypts is illustrated. Hematoxylin and eosin staining, 10× objective. Gastrocentro Archive, UNICAMP.

Among the lesions with an initial diagnosis of HP (*n* = 287), 172 (59.9%) maintained this diagnosis, 38 (13.3%) lesions were reclassified as SSL (Figure 1), and 77 (26.8%) received an unexpected diagnosis (tubular adenoma, inflammatory polyp, colonic mucosa with chorionic edema, or nonspecific inflammatory process).

**Figure 2:**
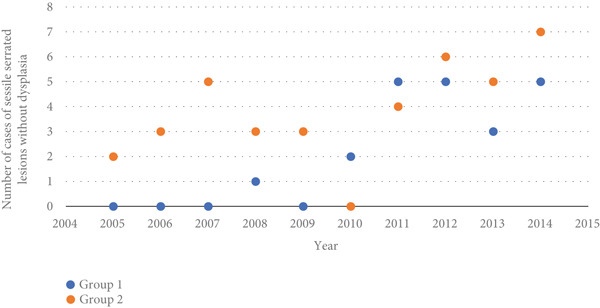
Number of cases of sessile serrated lesions without dysplasia diagnosed by group and year.

No dysplasia was found in the examined lesions. Diagnosis changes from HP to SSL occurred in 38 lesions and resulted in a 180% increase in the number of SSLs. Except for 2010, there was an increase in the total number of SSLs following histological review. In the years 2005, 2006, 2007, and 2009, there were no cases of SSL in Group 1 (Figure 2).

### 3.3. Demographic Data of SSL

#### 3.3.1. Sex and Age

The mean age of the individuals diagnosed with SSL was 63 (38–83) years, and 29 (50.9%) were female.

#### 3.3.2. Smoking

Twenty‐eight (49%) of the 59 patients diagnosed with SSL were active smokers or had a history of smoking; there were no data regarding two patients.

#### 3.3.3. Size, Morphology, and Location

Regarding size, 42 SSLs (71.2%) were < 10 mm, and 29 of these (49.2%) were diagnosed after histological reappraisal. Seventeen lesions (28.8%) were ≥ 10 mm; nine of these (15.5%) were diagnosed after histological review. There was no statistically significant difference between the number of SSLs < 10 mm diagnosed in the first examination and after histological review (*p* = 0.24).

Forty‐five (76.3%) SSLs were sessile, 13 had an elevated surface, and one was unclassified. Regarding location, 15 (25.4%) SSLs were found in the cecum, 25 (42.4%) in the ascending colon, and 19 (32.2%) in the transverse colon.

#### 3.3.4. Association With Conventional Adenomas

The presence of concomitant adenomas was detected in 22 (38.6%) examinations of patients with lesions diagnosed as SSL, with one adenoma being diagnosed in 12 (21%) exams, two adenomas in seven (12.3%) exams, three adenomas in two (3.5%) exams, and only one (1.7%) exam revealing more than three adenomas.

#### 3.3.5. Association With Serrated Lesions

Two (3.5%) patients had two SSLs in the same exam. HP was diagnosed concomitantly with SSL in 11 (19.3%) examinations, and in seven (63.6%), they were located in the proximal colon.

#### 3.3.6. Association With Colorectal Cancer

A history of colorectal cancer occurred in 14 (24.6%) individuals, and another four (6.8%) had colorectal adenocarcinoma concomitant with the diagnosis of SSL.

### 3.4. Interobserver Agreement

**Figure 3:**
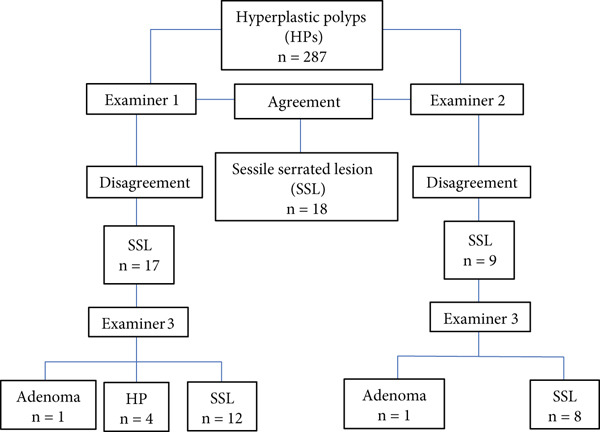
Distribution of the histological review among examiners and the results when the lesion was reclassified as SSL.

There was agreement between Examiners 1 and 2 regarding the diagnosis of SSL in 18 lesions of the 287 HPs submitted to histological review (Kappa 0.52, moderate agreement) and disagreement in 26 lesions (Examiner 1 diagnosed SSL in 17 lesions and Examiner 2 diagnosed HP in those lesions and Examiner 2 diagnosed another nine lesions as SSL while Examiner 1 diagnosed these 9 as HP). Examiner 3 assessed the 26 lesions in disagreement, and there was agreement with Examiner 1 regarding the diagnosis of SSL in 12 lesions (Kappa −0.19, no agreement/random agreement) and with Examiner 2 regarding the diagnosis of eight lesions (Kappa 0.13, poor agreement). Of the six lesions that were not reclassified as SSL, four had the diagnosis of HP maintained and two received the diagnosis of adenoma (Figure 3).

### 3.5. Immunohistochemistry

**Figure 4:**
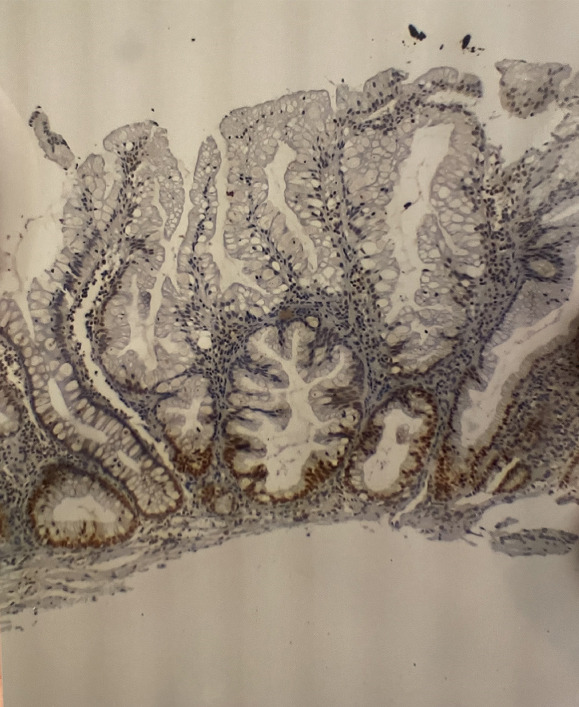
Immunohistochemical slide. Aspects of MLH1 protein expression, 10× objective. Gastrocentro Archives.

Of the 59 lesions diagnosed as SSLs, 21 were excluded due to the lack of suitable material for analysis. Ten paraffin blocks were not found, and 11 blocks did not have enough material to prepare new slides for analysis. All 38 lesions analyzed by immunohistochemistry demonstrated MLH1 expression. Cells with a large amount of mucus falsely appeared to have unstained nuclei, that is, were negative for MLH1 when examined at low magnification. The insets show the presence of staining in the evaluated areas (Figure 4).

### 3.6. Review of Missed Diagnosis of Dysplasia

Serrated and conventional dysplasia was not found in the histological review of the 59 lesions candidates for immunohistochemical analysis. Despite the absence of a diagnosis of dysplasia, five lesions stood out due to the presence of atypia. These anomalies were focally present up to the lower two‐thirds of the mucosa; cell maturation remained preserved toward the surface, and there was no sudden change relative to the adjacent SSL. All five lesions measured > 5 mm on the slide, and four (80%) measured ≥ 10 mm according to the endoscopist′s description. These lesions had both architectural and cytological atypia present in the same location.

The following architectural atypia were observed: focal side‐by‐side arrangement of crypts with little or no serration; skewed, curved, branching crypts, without angles; presence of well‐formed villi; and marked elongation of the crypts. Aspects regarding cytological atypia were enlarged nuclei, with moderate changes in shape and loss of basal polarity in small areas; cuboidal epithelial cells, with increased nucleus/cytoplasm ratio due to increased nuclear volume and loss of cellular secretion; decreased number of goblet cells, many of these dystrophic; epithelium varying from cuboidal to columnar in the same crypt, with loss of secretion; cellular hypersecretion; and increased mitoses, which were easily identified.

## 4. Discussion

The discovery of serrated lesions and their relationship with the development of colorectal cancer has brought new challenges. Various histological classifications of these lesions have been proposed in a short period of time, which may have contributed to missed diagnoses and, thus, differences in the prevalence and other epidemiological characteristics of serrated lesions.

Since SSLs occur more frequently in the proximal colon [7, 18–21], we chose to evaluate lesions in this location; most SLSs (67.8%) were found in the most proximal segments, both in the initial diagnosis and after reappraisal, with 15 (25.4%) and 25 (42.4%) lesions occurring in the cecum and ascending colon, respectively. Moreover, the morphology of the lesions was not different from that described in the literature; most (76.3%) were sessile lesions with no pedunculated lesions [7, 8, 18, 19, 22, 23].

The size of SSLs is a point of disagreement in the literature. While some authors demonstrate that these lesions are on average 6 mm in size, others claim that they are usually larger (around 20% larger), with some even reporting that most SSLs are bigger than 10 mm [7, 19, 20, 22, 23]. The comparison of lesion size after histological review did not show statistically significant differences in this series; thus, the hypothesis that smaller lesions were more difficult to diagnose (in general and in the first analysis) when there was less knowledge about SSLs was not confirmed.

Of the 308 lesions included in this study, there was a 180% increase in the diagnosis of SSLs after the reappraisal, and the diagnosis of 13.3% of HPs changed to SSLs. The fact that the diagnosis of most HPs was maintained confirms the data that HPs also occur in the proximal colon, although much less frequently than in the distal colon and rectum [7, 8, 18, 24]. As shown in Figure 2, there was an increase in the total number of SSLs diagnosed after histological review in the following years of this study since 2010, a 175% increase over the previous 5 years. The influence of the quality of the examined material is well recognized and widely discussed in the literature; well‐oriented crypts are required to assess full crypt extent and thus differentiate SSL from HP [2, 3, 7, 8, 18]. The use of more than one crypt for the diagnosis of SSL was applied by many pathologists; however, in 2019, new guidelines for SSL diagnosis—published by the WHO in 2010—eliminated this prerequisite [7, 8].

The analysis of the agreement between pathologists helps to validate the data of the study regarding the HP diagnostic changes to SSL. However, in this study, the low prevalence of the lesions interfered with the results of agreement between Examiners 1 and 3 and Examiners 2 and 3; the high agreement regarding the diagnosis of SSL did not lead to a satisfactory Kappa value in these cases. Therefore, it was not possible to determine the real agreement between Examiner 3 and Examiners 1 and 2.

Farris et al. [25] analyzed the interobserver agreement for distinguishing between SSA and HP. In their study, polyps < 5 mm were excluded, and five pathologists performed two reviews each; they also had a meeting to define which findings would be favorable to the diagnoses before the second reappraisal. The initial finding was moderate agreement in the diagnosis of SSA, which was maintained in the second reappraisal.

In their 2015 review article, Bateman and Shepherd [24] stated that the difficulty in diagnosing these lesions lies partly in the varying results among groups of pathologists. A few years earlier, Rex et al. [18] also stated in another review article that there was great variation in interobserver agreement in the diagnosis of these lesions, even among experienced pathologists, and that it was generally moderate to substantial. In 2019, Allende et al. [26] reported that it was still difficult to distinguish between SSAs and HPs regarding histological diagnosis. They assessed the interobserver agreement regarding these lesions and attempted to find stromal changes that would help in the diagnosis of SSAs. Using the Kappa value, they classified the agreement as moderate; additionally, the number of lesions was smaller than that in the present study.

In the present study, although there was an increase in the total number of SSLs diagnosed after the histological review, it was not possible to determine an association between the increase in the number of diagnoses and the accumulated knowledge, as no data were collected on the number of examinations performed in that period; determining the prevalence of these lesions over the years was hindered.

After the review by the examiners with good results, as demonstrated by the Kappa values, most diagnoses of HP in this study were maintained; this allowed stating that HPs are found in the proximal colon and provided evidence to corroborate the hypothesis that some HPs are precursor lesions for SSLs [8, 9, 27–30]. The microvesicular subtype is currently considered the potential lesion. The database used for this research did not mention the subtype of HPs included in the study. Therefore, it was not possible to identify whether the lesions that were reclassified as SSL had previously been classified as the microvesicular subtype, a hypothesis that, if confirmed, would corroborate the theory that the microvesicular subtype is the precursor lesion of the SSL.

The *BRAF* gene mutation found in these types of HPs is the main argument for this hypothesis, as it is found in more than 50% of SSLs and in up to 70%–80% of HPs belonging to this subtype [7–9, 13, 18, 24, 31–34]. Increased susceptibility to hypermethylation of cytosine/guanine islands, and therefore, the CIMP phenotype, is more commonly identified in HPs of the microvesicular subtype found in the right colon [7, 27, 31, 35]. Thus, suppression of *MLH1* gene expression is the differentiating molecular factor between HP and SSL. Although not all SSLs have this alteration, some SSLs may present a lack of *MLH1* expression becoming SSLD and rapidly developing into cancer [10, 36, 37].

There was no suppression of the MLH1 protein in the analyzed lesions, and dysplasia was not diagnosed which became a limitation in relation to the objective of this study. The absence of dysplasia can be explained by the rarity of this finding, although there is a wide variation in its prevalence in the current literature [20, 38, 39]. A higher prevalence of dysplasia in SSLs is found in studies conducted in specialized centers after cases are reviewed [14, 20, 39, 40].

In the latest WHO publication, the guidelines for diagnosing dysplasia in serrated lesions are concise and present few images, which are not helpful to less experienced pathologists. Moreover, this publication does not indicate the use of immunohistochemistry to aid diagnosis [8].

Liu et al. [14] conducted a thorough study wherein they define subtypes of serrated dysplasia. They recommend analyzing MLH1 suppression using immunohistochemistry in cases of doubt regarding the diagnosis of dysplasia and present detailed definitions and numerous images to guide the diagnosis.

Geramizadeh et al. [41] analyzed the suppression of MLH1 expression in all types of serrated lesions in a cohort from a single center and found no alterations, apart from three cases in the sample that had already been diagnosed with dysplasia. Based on these findings, they also recommend the criteria of the absence of MLH1 expression when the diagnosis of dysplasia is equivocal. Despite the publications by Liu et al. [14] in 2017 and the WHO publication in 2019 [8], the diagnosis of serrated dysplasia remains challenging as it has a variety of presentation patterns.

Therefore, the histological analysis of these lesions and the interobserver agreement were limitations of this study that should be considered.

Regarding the involvement of the serrated pathway in carcinogenesis, it remains unclear whether all microvesicular HPs progress to SSLs and which of these evolve to dysplasia. To date, there have been no findings regarding HP other than those already mentioned. The literature on progression to dysplasia posits the hypothesis that suppression of the *MLH1* gene first occurs in SSLs; progression to dysplasia occurs thereafter [8–10, 42, 43].

Five of the lesions analyzed in the present study had cytological and architectural changes that were not consistent with dysplasia. The absence of MLH1 expression in these lesions would indicate that they were advanced and that there were likely sufficient findings in some part of the lesion to diagnose dysplasia. Moreover, considering that these lesions were bigger than 10 mm in the endoscopist′s assessment, most were probably progressing to dysplasia when they were resected. Four lesions were diagnosed in the first examination and were thus not reviewed, which also supports the hypothesis that more advanced lesions are easier to identify. The small number of lesions with the aforementioned characteristics did not answer these questions, providing only postulations. Increased knowledge regarding the histology of SSLs can aid in their correct diagnosis and differentiation from HPs. Immunohistochemical analysis of MLH1 protein suppression is easy to perform and reproducible; however, it should only be used to help diagnose the occurrence of dysplasia. There are still gaps in the knowledge to be filled regarding the progression of serrated lesions considered benign to potentially malignant, as well as the predisposing or triggering factors for this transformation.

## 5. Conclusion

Changes in diagnosis from HP to SSL occurred in 13.3%. No dysplasia or lack of MLH1 expression was observed.

## Conflicts of Interest

The authors declare no conflicts of interest.

## Author Contributions

Priscilla de Sene Portel Oliveira conceived and designed the analysis, collected data, performed the analysis, and wrote the paper. Miriam Aparecida da Silva Trevisan contributed to the pathological analysis and manuscript review. Rita Barbosa de Carvalho contributed to the pathological analysis. Rita de Cássia Perina Martins contributed to the pathological analysis. João José Fagundes contributed to the preparation of the project and critical review. Claudio Saddy Rodrigues Coy coordinated the project from start to review.

## Funding

The study is supported by the Centre of Diagnosis of Digestive System Diseases (Gastrocentro), School of Medical Sciences, Universidade Estadual de Campinas (13083‐878). Gastrocentro financed the purchase of reagent material to carry out immunohistochemical studies.

## Data Availability

The data that support the findings of this study are available from the corresponding author upon reasonable request.
